# Increased Levels of VCAM-1 in Patients with High Cardiovascular Risk and Obstructive Sleep Apnea Syndrome

**DOI:** 10.3390/biomedicines12010048

**Published:** 2023-12-24

**Authors:** Ioana-Maria Chetan, Ștefan Cristian Vesa, Bianca Domokos Gergely, Ruxandra Stefana Beyer, Raluca Tomoaia, Georgiana Cabau, Damiana Maria Vulturar, Dana Pop, Doina Todea

**Affiliations:** 1 Department of Pneumology, “Iuliu Hatieganu” University of Medicine and Pharmacy, 400012 Cluj-Napoca, Romania; mariaioana_25@yahoo.com (I.-M.C.); biancadomokos@yahoo.com (B.D.G.); damiana_vulturar@yahoo.com (D.M.V.); doina_adina@yahoo.com (D.T.); 2 Department of Pharmacology, Toxicology and Clinical Pharmacology, “Iuliu Hatieganu” University of Medicine and Pharmacy, 400012 Cluj-Napoca, Romania; 3 Heart Institute “Nicolae Stancioiu”, 400001 Cluj-Napoca, Romania; anda_bogdan@yahoo.com; 4 Department of Cardiology, “Iuliu Hatieganu” University of Medicine and Pharmacy, 400012 Cluj-Napoca, Romania; raluca.tomoaia@gmail.com (R.T.);; 5 Department of Medical Genetics, “Iuliu Hatieganu” University of Medicine and Pharmacy, 400012 Cluj-Napoca, Romania

**Keywords:** cardiovascular risk, obstructive sleep apnea syndrome, VCAM-1, ICAM-1, epicardial fat thickness, SCORE2, SCORE-2OP

## Abstract

(1) Background: Although obstructive sleep apnea (OSA) is associated with increased cardiovascular morbidity, the link between OSA and cardiovascular disease (CVD) is not completely elucidated. Thus, we aim to assess cardiovascular risk (CVR) using SCORE 2 and SCORE 2 for older persons (SCORE 2OP), and to evaluate the association between the endothelial biomarkers VCAM-1, ICAM-1, epicardial fat, and sleep study parameters in order to improve current clinical practices and better understand the short-and long-term CVRs in OSA patients. (2) Methods: 80 OSA patients and 37 healthy volunteers were enrolled in the study. SCORE2 and SCORE 2 OP regional risk charts (validated algorithms to predict the 10-year risk of first-onset CVD) were used for the analysis of CVR. Two-dimensional echocardiography was performed on all patients and epicardial fat thickness was measured. VCAM-1 and ICAM-1 serum levels were assessed in all patients. (3) Results: OSA patients were classified as being at high CVR, regardless of the type of score achieved. Increased EFT was observed in the OSA group. VCAM-1 was associated with a high CVR in OSA patients, but no significant correlation was observed between adhesion molecules and epicardial fat thickness. (4) Conclusions: OSA patients have a high CVR according to the SCORE 2 and SCORE 2OP risk scores. VCAM-1 may be associated with a high CVR in OSA patients. Extending conventional risk stratification scores by adding other potential biomarkers improves the risk stratification and guide treatment eligibility for CVD prevention in the OSA population.

## 1. Introduction

Obstructive sleep apnea (OSA) is defined by the intermittent obstruction, either complete or partial, of the upper airway during sleep. These events lead to nocturnal intermittent hypoxia, sleep fragmentation, daytime sleepiness, and snoring [1]. OSA is estimated to affect nearly 1 billion people worldwide. Historically, OSA was considered a disorder affecting predominantly obese males, but it is presently recognized that a significant portion of OSA patients are women, and a normal body mass index (BMI) is often related to it [2]. Multiple studies have shown that OSA is correlated with increased cardiovascular morbidity [3,4]. Moreover, affected patients are unaware of disturbances in their sleep, and most are diagnosed due to related cardiovascular comorbidities, such as coronary artery disease, arterial hypertension, atrial fibrillation, stroke, or heart failure [4,5].

The link between OSA and cardiovascular diseases (CVD) is not completely elucidated, but most reports point towards autonomic, metabolic, hemodynamic, and inflammatory dysregulations caused by the subsequent hypoxia resulting from an obstructive sleep pattern [2]. It is presently recognized that atherosclerosis is a chronic inflammatory disorder, and it has been shown that OSA-induced hypoxia accelerates the process of atherogenesis [1]. In the early stages of atherosclerosis, the activated endothelium recruits circulating immune cells to the site of inflammation by releasing cytokines and cellular adhesion molecules, particularly intercellular adhesion molecule-1 (ICAM-1) and vascular cell adhesion molecule-1 (VCAM-1), facilitating vascular permeability and leukocyte migration [6]. The initial sign of disease activity in animal and human models of atherosclerosis is marked by an increase in adhesion molecules. In a recent review, Fiedorczuk et al. emphasized the role of adhesion molecules by characterizing both their similarities and differences. ICAM-1 is a glycoprotein ranging from 80–110 kDa and serves as a ligand for lymphocyte-function-associated antigen-1. VCAM-1 belongs to the immunoglobulin superfamily of adhesion molecules [7]. ICAM-1 is persistently expressed in the membranes of leukocytes and endothelial cells, whereas VCAM-1 is expressed in the membranes of vascular endothelial cells only if it has been stimulated by cytokines [8]. In terms of which adhesion molecule plays a crucial role in the development of atherosclerosis and, implicitly the occurrence of CVD, the studies published to date present contradictory results. On one hand, elevated circulating levels of both ICAM-1 and VCAM-1 were shown to be associated with increased cardiovascular risk, acute coronary syndromes, and atherosclerotic disease [9]. On the other hand, there are published data supporting the dominant role of VCAM-1 in the pathogenesis of atherosclerotic lesions [10,11]. Therefore, it remains to be seen what the exact pathophysiological mechanism is, and which adhesion molecule has the greatest negative impact.

Another observation concerning patients with OSA is an increase in epicardial adipose tissue (EAT) [12,13], which can be assessed using an echocardiography [14,15]. The epicardial fat thickness (EFT) is independently shown to contribute to the pathogenesis of CVD by releasing pro-inflammatory and pro-atherogenic cytokines, with both OSA and EAT being associated with an increased cardiometabolic risk [16].

The cardiovascular risk assessment of the OSA population is essential for the prevention of adverse cardiovascular events. Recent data show that OSA increases the risk of heart failure by 140% and coronary artery disease by 30% [17]. In a recent paper, Kim et al. evaluated the association between carotid intima-media thickness (IMT) as a cardiovascular risk factor for OSA and the Framingham risk score (FRS), which is a specific algorithm used to estimate the 10-year cardiovascular risk of an individual. Increased IMT in the severe OSA group could not be completely explained with classical risk factors defined with the FRS, suggesting that the FRS might be insufficient to determine the risk accurately [18].

Risk scores, such as the systematic coronary risk evaluation 2 (SCORE2) and the systematic coronary risk evaluation 2 for older persons (SCORE 2OP), can be useful. SCORE2 is an updated algorithm specifically designed for the European population to predict the 10-year risk of first-onset CVD. The difference between the two risk scores is that SCORE 2OP estimates incident cardiovascular event risks in older patients (70–89 years old), while SCORE2 is for the younger population (40–69 years old) [19,20].

An appropriate CVR assessment is yet to be defined in the OSA population, with the research to date lacking complete data regarding the use of cardiovascular risk scores for these patients. An improvement in the predictive accuracy of cardiovascular risk scores, potentially by incorporating specific parameters, like VCAM-1, ICAM-1, EFT, and sleep study variables, would be necessary to comprehensively assess the short- and long-term risks of OSA before integrating them into medical practices.

In this study, we aim to assess cardiovascular risk using SCORE2 and SCORE2OP to evaluate the association between the endothelial biomarkers VCAM-1, ICAM-1, epicardial fat, and sleep study variables in order to improve the current clinical practice, and better understand the short- and long-term cardiovascular risks in patients with OSA.

## 2. Materials and Methods

### 2.1. Study Population

From January 2020 to February 2021, we conducted a screening of 163 adult patients admitted to the “Leon Daniello” Pneumology Hospital of Cluj-Napoca for OSA. The study complied with the principles of the Declaration of Helsinki and was approved by the Ethics Committee for Human Research (number: 493/2019). Prior to participation, all the patients provided informed consent.

The patients were included in the study if they were ≥40 years old and had at least 3 clinical manifestations of OSA, including snoring, witnessed apneas, episodes of gasping or choking, daytime sleepiness that could not be attributed to other factors, nocturia, morning headaches, and a lack of concentration and memory.

Exclusion criteria comprised patients with decompensated or unstable cardiopulmonary conditions, chronic respiratory diseases, like chronic obstructive pulmonary disease (COPD), sarcoidosis, and idiopathic pulmonary fibrosis (IPF). In addition, subjects with a history of cor pulmonale, documented pulmonary embolism, significant right- or left-sided valvular heart diseases, known cardiomyopathies, ischemic heart disease, congenital heart disease, pericardial disease, diabetes mellitus, malignancy, psychiatric disorders, or recent surgery were not included.

**Figure 1:**
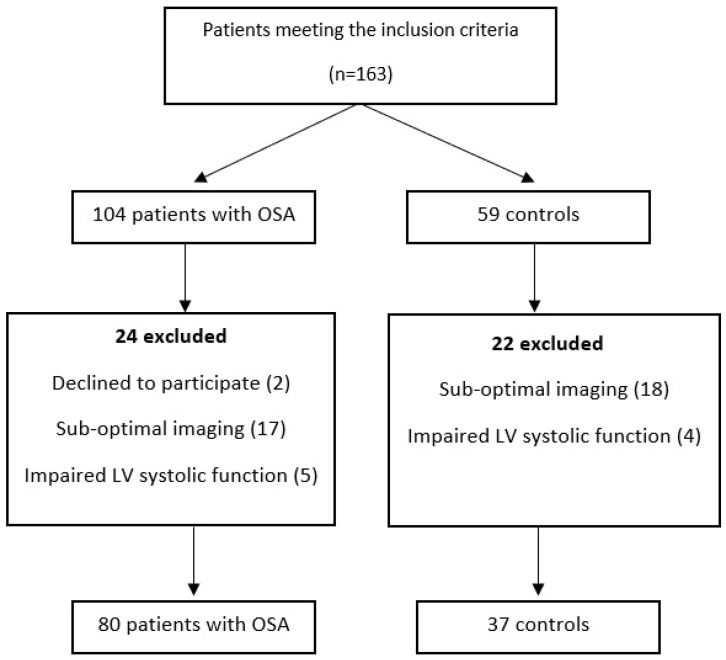
Study flowchart.

We ultimately enrolled 117 participants who were divided into 2 groups. The study group consisted of 80 patients who were clinically diagnosed with OSA. The healthy volunteer group was composed of 37 age- and sex-matched individuals who did not meet the criteria for OSA according to the respiratory polygraphy report. The OSA patients were not undergoing current treatment with continuous positive airway pressure (CPAP) or bilevel positive airway pressure (BIPAP) (Figure 1).

The cardiovascular risk profile was evaluated during the initial appointment. This included personal pathological history, medication use (antihypertensive agents and lipid lowering drugs), smoking status, blood pressure, electrocardiogram, transthoracic echocardiography, a sleep study, physical exam, blood exam, and estimations of the 10-year risk of fatal and non-fatal cardiovascular diseases (CVDs) using SCORE2 and SCORE2-OP regional risk charts.

Patients who smoked at least one cigarette in the past month were categorized as current smokers. Patients who reported a history of hypertension and had consistently taken antihypertensive drugs in the previous month were categorized as having pre-existing systemic arterial hypertension.

### 2.2. Sleep Study

All enrolled patients underwent a cardiorespiratory sleep study with the Nox T3 polygraph during a whole night. The sleep examination included the continuous monitoring of nasal pressure, activity and heart rate, oxygen saturation by pulse oximetry, tracheal sounds (microphone), rib cage and abdominal movements, as well as body position. Trained personnel carefully analyzed and confirmed the results of the sleep evaluation. Apnea was described as a complete cessation of airflow lasting a minimum of 10 s. Hypopnea was defined as a reduction in airflow of at least 50% lasting for at least 10 s. Obstructive apnea was defined as apnea associated with paradoxical thoracic and abdominal movements [21]. The total number of apneas and hypopneas was referred to as the apnea–hypopnea index (AHI). OSA was diagnosed if the AHI was greater than or equal to 5 events per h of sleep, with documented symptoms of daytime sleepiness, insomnia, unintended sleep episodes while awake, emotional disorders, snoring, respiratory disturbances, or a history of cerebrovascular events, high blood pressure, or coronary artery disease. Patients with an AHI ranging from 5 to 15 events per h of sleep were considered to have a mild form of OSA, patients with AHIs between 15 and 30 events per h of sleep were considered moderate, and those with AHIs of 30 or more were classified as having severe OSA [21,22].

### 2.3. Echocardiography

**Figure 2:**
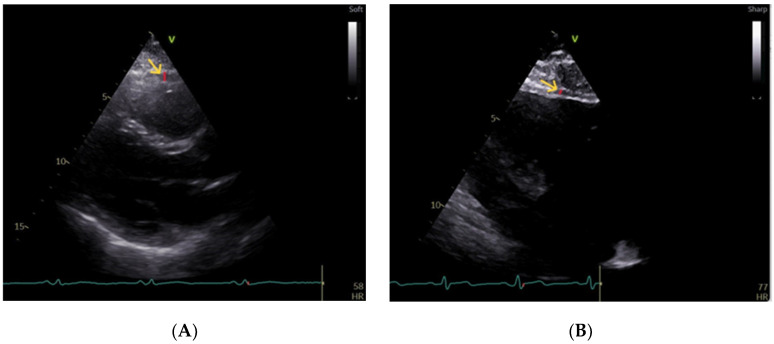
Epicardial fat thickness measurement by echocardiography in the parasternal longitudinal view (green arrow indicating the area of interest). Epicardial fat (indicated by red line and yellow arrow) with increased thickness in a patient with OSA (**A**) and minimum epicardial fat in a healthy volunteer (**B**).

Echocardiographic data were obtained for all the participants. A standard two-dimensional (2D) transthoracic echocardiography and Doppler evaluation were performed on a Vivid E95 scanner (GE Vingmed Ultrasound, Horten, Norway). The standard echocardiographic measurements were performed using a 2D matrix array (M5S) according to the latest recommendations [23]. The left ventricular ejection fraction (LVEF) was determined through the manual tracing method (biplane Simpson’s). Epicardial fat thickness appeared as a hypoechoic space situated between the visceral layer of the pericardium and the outer wall of the myocardium. The maximum thickness was measured perpendicular to the free wall of the right ventricle (RV) in the standard parasternal long axis view at the end of systole in three cardiac cycles [24]. An example of an echocardiogram image is shown in Figure 2.

### 2.4. Measurements of Anthropometric and Biochemical Parameters

Fasting blood samples were obtained from all patients to determine the levels of intercellular adhesion molecule-1 (ICAM-1) and vascular cell adhesion molecule 1 (VCAM-1), triglycerides, C-reactive protein (CRP), glycemia, and non-HDL-C. The venous blood samples were collected upon admission and were centrifuged at 3500–4000× *g* rpm for 15 min to obtain the serum, which was aliquoted for storage in Eppendorf tubes. Subsequently, the samples were stored in a freezer at −80 °C. Both ICAM-1 and VCAM-1 serum levels were diluted and the concentration was measured by a sandwich enzyme-linked immunosorbent assay (ELISA), according to the manufacturer’s instructions for the assays (Quantikine ELISA Human ICAM-1/CD54 Non-Allele-specific, Human VCAM-1/CD106, R&D Systems, Minneapolis, MN, USA). All the samples measured fell within the range of values of the standard curve. Values were expressed as ng/mL. The rest of the measurements were analyzed in accordance with standard laboratory methods.

Body weight and length were assessed and documented. Body mass index (BMI) was calculated using the formula body weight/height^2^ (kg/m^2^). All measurements were performed by the same person using standardized instruments. Obesity was characterized by a body mass index (BMI) ≥ 30 kg/m^2^.

### 2.5. Cardiovascular Risk Stratification Using the Scores

The following tools were used to analyze cardiovascular risk in this study: SCORE2 (“systematic coronary risk evaluation”, aged 40–69 years) and SCORE2 OP (“older persons”, aged 70–89 years) regional risk charts developed by the European Society of Cardiology (ESC) [19,20]. These allowed the estimations of the 10-year risk of fatal and non-fatal cardiovascular diseases (CVDs) in people without previous CVD or diabetes based on gender, age, systolic blood pressure, non-HDL cholesterol, and smoking status. A different risk table must be used depending on the country; in our case, we used the table for very high overall risk of cardiovascular disease, since Romania is included in this category of CVR. In terms of CVR analyzed using these scores, the participants were classified as low, intermediate, or high risks according to Table 1 [19,20].

### 2.6. Statistics

A statistical analysis was performed using MedCalc^®^ Statistical Software version 19.7 (MedCalc Software Ltd., Ostend, Belgium; https://www.medcalc.org; accessed on 6 May 2023). Quantitative data were tested for normal distributions using the Shapiro–Wilk test and expressed as median and 25–75 percentiles. Categorical data were expressed as frequency and percentage. The chi-squared test was used to test for differences in frequency, while the Mann–Whitney test was used to compare continuous variables between the two groups. The correlation between variables was assessed using the Spearman correlation coefficient. To account for potential cofounders, we performed logistic regression analyses to identify any significant relations between age and sex and circulating adhesion molecules VCAM-1 and ICAM-1. ROC curves were used to obtain cut-off values for biomarkers in relation to CVR. A *p*-value of less than 0.05 was considered statistically significant.

## 3. Results

### 3.1. Baseline Characteristics

Table 2 provides a comprehensive overview of the baseline characteristics of both groups. No statistically significant differences were found in the prevalence of gender between the two groups. The median BMI of the patients with OSA tended to be higher than the BMI of the controls, although all participants showed BMI levels above the healthy weight range. When evaluating the clinical characteristics, 33.8% of the study group were smokers, with 45.9% in the control group. Regarding the presence of systemic arterial hypertension, there was a significant difference between the two groups, with patients with OSA showing a higher prevalence compared to the controls (91.3% vs. 59.4%).

For intercellular adhesion molecules, we observed higher circulating levels of VCAM-1 in patients with OSA compared to the controls (*p* < 0.001) with no difference for ICAM-1 between the two groups (*p* = 0.865). Furthermore, CRP levels were higher in the OSA group compared to the controls (*p* < 0.001).

OSA patients tended to have significantly increased epicardial fat thickness (*p* < 0.001) compared to the controls.

The median value for cardiovascular predicted risk classifies the OSA population as being at a high risk, regardless of the type of score used (SCORE2 or SCORE2-OP). When assessing SCORE-2 or SCORE-2 OP, in terms of predicting the 10-year risk of first-onset CVD in the study population, the highest percentage of OSA patients was classified as high risk (85%), while in the control group, the low/intermediate risk class had the highest percentage.

### 3.2. Association between Serum Biomarkers and Epicardial Fat with CVR Scores

Table 3 details the relations between the CVR scores and serum biomarkers, sleep study parameters, and EFT among OSA patients. When correlating the values of the assessed parameters with the risk stratification categories, we observed that a statistically significant association was found between the CVR classes and VCAM-1. In particular, OSA patients with higher CVRs had higher serum VCAM-1 levels. The model did not change when it was adjusted for potential confounding factors. The results of multiple logistic regression analyses, including age and sex, are summarized in Table 4. Multiple regression analyses demonstrated that VCAM-1 was associated with high CVR outcomes independent of age or sex. No statistically significant correlation was found for the other parameters.

Table 5 shows the correlations between adhesion molecules and EFT as CVR factors in the two groups. No significant correlation was observed between these parameters. Although there was not a statistically significant association between VCAM-1 and EFT in the OSA group, the *p*-value of 0.06 was possibly important in practice.

Table 6 shows the comparison between the ROC curves of serum biomarkers and sleep parameters, which was performed to evaluate the predictive abilities of these parameters in discriminating the high-CVR category. There was no significant difference between the AUCs. The only variable that became statistically significance in the ROC analysis was VCAM-1 (*p* = 0.03). The cut-off value for VCAM-1, above which it was associated with an increased risk, was 1904 ng/mL (Se 80.88% (95%CI: 69.5–89.4); Sp 66.67% (95% CI: 34.9–90.1).

## 4. Discussion

The present study, based on our information, is the first to investigate the relationship between serum biomarkers VCAM-1 and ICAM-1, EFT, and the CVR predicted by SCORE-2 and SCORE-2OP risk tools in patients with obstructive sleep apnea. Our study revealed that a significant proportion of OSA patients had an increased CVR based on the SCORE-2 and SCORE-2 OP risk scores. Furthermore, we observed a remarkable increase in serum VCAM-1 levels in OSA patients, especially in those with a high CVR as assessed by the two risk scores.

OSA has been linked to various cardiovascular diseases. Numerous studies have shown a robust correlation between sleep disorders and cardiovascular pathology, irrespective of the common risk factors [3,4]. More than 50% of patients with OSA have systemic blood pressure, and approximately a quarter of patients with systemic blood pressure have OSA [25]. Many retrospective and cross-sectional studies have demonstrated a greater risk of coronary artery disease in OSA. In angiographically verified coronary artery disease, 30% of women and 37% of men had OSA [1]. Pathophysiologic mechanisms proposed to explain this relationship include oxidative stress, sympathetic nervous system dysfunction, and endothelial dysfunction [26,27].

Despite substantial advances in reducing the global burden of CVD through addressing traditional risk factors, a notable residual risk persists, and low-grade inflammation emerges as one of the most influential risk modifiers. [28]. Atherosclerosis, the leading risk factor for CVD, is a low-grade chronic inflammatory condition linked to lipid intensification within the vascular system and subsequent plaque formation [29]. It has been proposed that increased cell adhesion molecule levels likely increase the risk of future cardiovascular events. Adhesion molecules (ICAM-1 and VCAM-1) facilitate the attachment of circulating leukocytes to the endothelium, facilitating their transmigration and accumulation in the arterial intima [30]. The expression of these molecules is a marker of endothelium inflammation and is thought to contribute to the initiation of atherosclerosis. Studies have supported their role in the development of CVD [31]. The precise mechanism of the development of cardiovascular disease in patients with OSA is not yet fully understood. Some studies suggest that the repetitive hypoxia caused by OSA may be involved in the pathogenesis of cardiovascular pathologies by stimulating inflammatory responses through increased levels of adhesion molecules and cytokines [32]. Both in vitro and in vivo studies have demonstrated an association between hypoxia and increased levels of adhesion molecules [1,33], while others do not support this hypothesis [34].

Several previous investigations have reported an association between OSA and elevated circulating levels of adhesion molecules. In a recent meta-analysis, it was shown that ICAM-1 levels were increased in OSA patients compared to the controls, and in accordance with OSA severity [35]. Ohga et al. determined circulating L-selectin, ICAM-1, and VCAM-1 levels in seven OSA patients and six age-matched controls pre- and post-sleep. They showed that, in comparison to the controls, OSA patients had higher levels of these biomarkers [36]. In another study, El-Solh et al. conducted a comparative analysis of age, gender, BMI, and severity of coronary heart disease between 15 individuals with moderate-to-severe OSA and a matched control group. The OSA group showed elevated levels of ICAM-1, VCAM-1, and E-selectin [37]. Similarly, Ursavas et al. found that individuals with moderate/severe OSA had higher levels of ICAM-1 and VCAM-1 than their healthy counterparts [1]. Our results are in concordance with other studies [38,39,40], demonstrating that VCAM-1 levels are increased in the OSA group compared to the controls, while ICAM-1 levels show no significant differences between the two groups. Notably, studies indicate that higher levels of ICAM-1, but not VCAM-1, are reliable predictors of CVR in initially healthy populations [38,39,40]. However, it is crucial to note that elevated VCAM-1 levels do not appear to pose a risk to healthy subjects in the absence of endothelial dysfunction. Instead, they are predictive indicators of future cardiovascular risks in patients with pre-existing conditions, such as diabetes, arterial hypertension, or cardiac ischemic disease [39,41]. A plausible explanation for this can be due to the variations in expression. ICAM-1 is expressed at steady-state levels by multiple cell types, while VCAM-1 is induced under pro-atherosclerotic conditions [8,39,42]. In light of the current evidence, it is reasonable to suggest that OSA is a pro-atherosclerotic disease [43]. Myron et al. further elucidated the significance of VCAM-1 in the initiation of atherosclerosis, despite the upregulation of both VCAM-1 and ICAM-1 expressions in atherosclerotic lesions [10]. These findings were reinforced by Cai et al. substantiating the role of VCAM-1 both in the beginning stages of atherosclerosis as well as its later stages [11].

In support to the existing knowledge, our investigation provided a novel contribution by establishing a cut-off value for VCAM-1. By defining a novel risk threshold, our study holds significant clinical implications for discerning individuals with obstructive sleep apnea and concomitant heightened cardiovascular risk. The determination of cut-off values for adhesion molecules, particularly VCAM-1, has already been proven beneficial for the detection of pathologies associated with atherosclerosis, such as peripheral arterial disease, atrial fibrillation, post-acute myocardial infarction, and heart failure [44,45,46]. Notably, Ursavas et al. defined significant levels of VCAM-1 and ICAM-1 predictive of OSA [1]. However, despite these valuable findings, there is a lack of comprehensive studies characterizing the levels of adhesion molecules associated with increased cardiovascular risks in patients with OSA. Compared to previous studies analyzing the levels of cell adhesion molecules in those who did or did not present CV events over several years [9,47], we showed that VCAM-1 was associated with a high cardiovascular risk predicted by the SCORE-2 and SCORE-2OP risk tools in OSA patients, supporting its use as a marker of CVR and bridging the gap in the knowledge.

The early initiation of OSA therapy can prevent future cardiovascular events, which is consistent with previous studies showing that unmanaged severe OSA increases the risk of cardiovascular events in long-term follow-up treatments. Appropriate CPAP usage limits the significant increase in ICAM-1 and VCAM-1 levels observed in non-CPAP users [48]. Pak et al. demonstrated in their research that the use of CPAP in individuals with moderate to severe OSA prevented the increase in adhesion molecules observed in non-users over a two-year period [49]. Another paper reported a significant reduction in ICAM-1 levels after 6 months of CPAP treatment [50]. The randomized controlled trial RICCADSA involved 210 OSA patients. The concentrations of circulating adhesion molecules were examined at the beginning and after one year of CPAP therapy. CPAP treatment was linked with a decrease in VCAM-1 levels and showed a trend towards significance in the reduction in ICAM-1 levels [51]. Finally, there are also other studies with smaller samples showing that the concentrations of adhesion molecules decrease with CPAP therapy [52,53].

In our study, we showed that CRP levels were increased in OSA group compared with the controls, underlining the presence of a proinflammatory status in this population. These results are in agreement with those of Shamsuzzaman et al. [54]., who demonstrates that OSA is associated with high CRP levels, and that they are directly proportional to the severity of the disease. Nevertheless, the elevated CRP levels in OSA patients could not be fully explained by the classical risk factors defined with SCORE2 and SCORE2 OP. One explanation for this may be that CRP influences OSA independently of other risk factors. These findings can be confirmed by other larger studies and RCTs where no changes in the CRP levels are observed after CPAP treatment [26,55]. Once again, CRP is only one facet of the complicated inflammatory process associated with OSA. If these biomarkers have a greater impact on OSA, independent of other risk factors, their influence on the estimation of CVR by the two scores may be limited.

Furthermore, in our study, we explored the relationship between EFT and cardiovascular risk predicted by SCORE-2 and SCORE-2OP. We observed that the mean epicardial fat thickness of OSA patients was significantly higher compared to the control group. These results are consistent with the study of Zhang et al., who demonstrated that EFT was positively correlated with OSA [56]. More recently, a meta-analysis study demonstrated that patients with OSA had higher EFT levels compared to patients without OSA, as measured by echocardiography. The increase in EFT correlated with the severity of OSA [14]. Additionally, there is published work that emphasizes the favorable impact of CPAP therapy on reducing EFT. Kostopoulos et al. demonstrated that three months of CPAP usage led to beneficial short-term modifications of EFT [57]. Similarly, in another study, Suha et al. achieved the same results, but CPAP therapy was used for a period of 24 weeks [58]. The exact mechanism underlying the observed reduction in EFT is not well understood. In addition, it is worth noting that, despite the association between EFT, atherosclerosis, and unfavorable outcomes, there are currently no data for the prognostic significance of EFT reduction that may be associated with improved outcomes [58,59]. Epicardial fat thickness is considered a cardiometabolic risk factor for OSA. Epicardial adipose tissue (EAT) encompasses the coronary arteries and secretes various cytokines, chemokines, and interleukins, which may be either harmful or protective based on the specific local conditions [60]. The expansion of EAT may determine the secretion of more proinflammatory molecules associated with cardiovascular disease and metabolic syndrome [61]. There have been reports indicating a correlation between the increase in EAT, the presence of OSA, and BMI. In a recent meta-analysis that first compared the EFT of OSA patients and controls with significantly different BMIs, the investigators found that the EFT was significantly increased in the OSA patients. They then compared the EFTs of OSA patients and controls with similar BMIs and found that the OSA patients also had greater EFTs than the controls [62]. These results are similar to those of our study, where the EFT was increased in the OSA group compared to the control group. The BMI values for the two groups were similar, indicating they were overweight. Additional studies demonstrated a strong correlation between OSA and EFT in obesity and non-obesity cases [13,63].

Although increased EFT may contribute to the incidence and progression of OSA by secreting proinflammatory cytokines [62], in our study, neither VCAM-1 nor ICAM-1 were associated with EFT in the OSA group, and additional EFT was not associated with a high CVR. The fact that increased EFT in the OSA group cannot be fully explained by the classical risk factors defined by SCORE2 and SCORE2 OP might imply that EFT affects OSA independently of other risk factors. These results may be due to the small sample size, although the possibility of being important in practice cannot be excluded.

However, the research of Aitken et al. [64] demonstrated a significant association between EFT and the upper body fat deposit index. EFT might indicate an increased deposition of ectopic fat in the upper airway, causing disordered breathing, but does not affect OSA by secreting proinflammatory cytokines.

Models that use risk factors to predict the likelihood of cardiovascular events are helpful in identifying high-risk OSA patients who may benefit from more extensive CV risk management. Archontogeorgis K et al. demonstrated in their study that the severity of OSA was associated with an increase in the 10-year risk for cardiovascular morbidity and mortality, as indicated by the Framingham risk score (FRS) and SCORE [65]. In our study, we showed that only VCAM-1 as a biomarker of endothelial dysfunction manifested higher levels in patients with a higher CV risk. Our observation contributes to the existing evidence in the general population and is consistent with several studies [66,67] that establish an association between cell adhesion molecules and CV events. This association is attributed in part to a robust association between proinflammatory status and CV risk factors.

## 5. Limitations

The most important limitation of the study was connected to its cross-sectional design, which prevented the establishment of a direct association between the parameters assessed and the CV outcomes. Furthermore, the generalizability of our results was limited to the population similar to that used in our study, as SCORE2 and SCORE2 OP were risk scores that were validated for predicting the 10-year risk of first-onset CVD in European populations. To exclude other potential sleep disorders, a polysomnography test would have been preferable, but was not feasible due to the cost and technical limitations. Moreover, this study was conducted at a single center with a limited number of patients, which could have led to sample bias. Future, prospective, long-term follow-up studies are essential to establish the meaningful discriminating power of both the biomarkers and applied risk scores in this population. However, the data presented in this research correlate the biomarkers, especially VCAM-1, with cardiovascular risks predicted by widely used scores in clinical daily practice. This area is an important subject to be investigated further.

## 6. Conclusions

In conclusion, we compared OSA patients with healthy individuals and found out that OSA patients had high CVR scores according to SCORE-2 and SCORE-2OP. In addition, our findings suggest that there is an association between VCAM-1 and high CVR as predicted by the two scores. This observation suggests the existence of endothelial dysfunction and atherosclerosis in the OSA population. Moreover, we defined a cut-off value for VCAM-1 that was associated with increased CVR. This could be clinically relevant in the identification of OSA patients with a high CVR. The results presented here indicate the necessity for extending conventional guideline-based risk stratification scores by adding novel biomarkers, such as VCAM-1, to further improve the risk stratification and guide treatment eligibility for cardiovascular disease prevention in the OSA population. It is important that the studied sample is monitored over time. This will facilitate the detection of future cardiovascular events and confirm the reliability of applied risk scores and biomarker assessments, particularly VCAM-1.

## Figures and Tables

**Table 1:** The predicted 10-year cardiovascular disease risks.

CVR	SCORE2		SCORE2-OP
	<50 Years	50–69 Years	≥70 Years
Low	<2.5%	<5%	<7.5%
Intermediate	2.5% to <7.5%	5% to <10%	7.5% to <15%
High	≥7.5%	≥10%	≥15%

**Table 2:** Baseline characteristics of all randomized patients.

Variable	Patients (n = 80)	Controls (n = 37)	*p*-Value
Age years	60 (51; 67)	55 (42; 62)	0.008
Sex female, n, %	34 (42.5%)	18 (48.6%)	0.673
BMI kg -m^2^	34 (30.8; 40.2)	29 (28.5; 30.1)	<0.001
Additional cardiovascular risk factors:			
-Arterial hypertension, n, %	63 (91.3%)	22 (59.5%)	<0.001
-Non-HDL Cholesterol, mg/dL	101.5 (82.5; 125.2)	101.0 (89.0; 123.0)	0.74
-Glycemia, mg/dL	109.0 (100.0; 123.0)	90.0 (85.0; 103.0)	<0.001
-Triglycerides, mg/dL	120.0 (95.0; 154.0)	98.0 (89.0; 120.0)	0.012
-Smoking, n, %	27 (33.8%)	17 (45.9%)	0.289
Results of the sleep study:			
AHI h^−1^	28.6 (18.3; 42.1)	2.8 (1.2; 3.3)	<0.001
ODI h^−1^	29.2 (21.9; 42.9)	3.5 (2.0; 6.2)	<0.001
Echocardiographic parameters			
LVEF, %	55 (50; 55)	55 (55; 63)	0.001
Epicardial fat, mm	6 (4.7; 7.0)	3 (1.5; 5.0)	<0.001
Serum parameters			
ICAM-1, ng/mL	120 (96.5; 139.5)	126 (83; 163)	0.865
VCAM-1, ng/mL	2160 (1896.5; 2407.5)	1820 (1625; 1906)	<0.001
CRP, mg/L	1.0 (0.60; 2.0)	0.20 (0.20; 0.30)	<0.001
SCORE2; SCORE2-OP			
Risk prediction, %	15.5 (10; 22.7)	7 (5; 13.5)	<0.001
Risk classification			
Low/intermediate risk	12 (15%)	25 (67.6%)	<0.001
High risk	68 (85%)	12 (32.4%)	<0.001

Data are presented as median (25–75 percentiles) and n (%); BMI: body mass index, AHI: apnea-hypopnea index, ODI: overnight desaturation index, LVEF: left ventricle ejection fraction, ICAM-1: intercellular adhesion molecule-1, VCAM-1: vascular cell adhesion molecule-1, CRP: C-reactive protein, SCORE2: systematic coronary risk evaluation 2, SCORE 2OP: systematic coronary risk evaluation 2 older persons.

**Table 3:** Relationship between cardiovascular risk scores and echocardiographic, sleep study, and serum parameters in OSA patients.

Parameters	Low/Intermediate Risk	High Risk	*p*-Value
ICAM-1, ng/mL	133.9 (92.6; 152.8)	119 (98.7; 138)	0.509
VCAM-1, ng/mL	1871 (1623; 2184)	2187 (1923.5; 2410)	0.03
CRP, mg/L	1.50 (0.70; 6.55)	1.0 (0.60; 2.0)	0.27
Non-HDL cholesterol, mg/dL	103.5 (100.25; 120.0)	100.5 (78.0; 128.0)	0.58
Glycemia, mg/dL	100.5 (93.5; 111.5)	110.0 (101.0; 128.0)	0.08
EFT, mm	6.1 (4.5; 8.1)	6 (5; 7)	0.589
AHI h^−1^	35.1 (20.4; 68.7)	29 (20.5; 42.0)	0.25
ODI h^−1^	34.5 (18.7; 74.4)	30.2 (22; 44.7)	0.45

Data are presented as median values (25–75 percentiles). ICAM-1: intercellular adhesion molecule-1, VCAM-1: vascular cell adhesion molecule-1, CRP: C-reactive protein, EFT: epicardial fat thickness, AHI: apnea–hypopnea index, ODI: overnight desaturation index.

**Table 4:** Multiple regression analysis of factors associated with high CVR.

Variables	OR	95% CI for OR		*p*-Value
		Lower	Upper	
VCAM-1 > 1904 ng/mL	6.533	1.307	32.662	0.022
Age	1.112	1.026	1.205	0.009
Sex: male	0.315	0.054	1.846	0.200

VCAM-1: vascular cell adhesion molecule-1, OR: odds ratio; CI: confidence interval.

**Table 5:** Correlation between epicardial fat thickness and serum biomarkers.

	Epicardial	Fat Thickness		
	OSA Patients		Controls	
	r	p	r	p
**ICAM-1**	0.19	0.86	0.22	0.18
**VCAM-1**	0.20	0.06	-0.98	0.50

OSA: obstructive sleep apnea, ICAM-1: intercellular adhesion molecule-1, VCAM-1: vascular cell adhesion molecule-1.

**Table 6:** Comparison of ROC curves.

Variables; AUC	Pairwise	Comparison	95% CI	*p*-Value
ICAM-1; 0.563	ICAM-1	AHI	0.0215 to 0.356	0.060
VCAM-1; 0.696		ODI	0.0298 to 0.360	0.059
AHI; 0.604		CRP	−0.0350 to 0.327	0.11
ODI; 0.568	VCAM-1	AHI	−0.123 to 0.128	0.97
CRP; 0.599		ODI	−0.117 to 0.134	0.89
		CRP	−0.0811 to 0.162	0.51

ROC: receiver operating characteristic curve, AUC: area under the curve, CI: confidence interval, ICAM-1: intercellular adhesion molecule-1, VCAM-1: vascular cell adhesion molecule-1, AHI: apnea–hypopnea index, ODI: overnight desaturation index, CRP: C-reactive protein.

## Data Availability

The datasets generated and/or analyzed in the current study are not publicly available due the fact that they constitute an excerpt of research in progress; but, they are available from the corresponding authors on reasonable request.
